# Valorization of Wheat Straw Cellulose into Biodegradable Packaging Films for Fresh Produce Preservation

**DOI:** 10.3390/foods15173111

**Published:** 2026-09-01

**Authors:** Sharad Bhattarai, Srinivas Janaswamy

**Affiliations:** Department of Dairy and Food Science, South Dakota State University, Brookings, SD 57007, USA; sharad.bhattarai@jacks.sdstate.edu

**Keywords:** wheat straw, valorization, biodegradable film, optimization, film characterization, fruit stability

## Abstract

The growing environmental impact of petroleum-based plastic packaging has accelerated the development of biodegradable materials from renewable resources. In this study, cellulose extracted from wheat straw was regenerated into biodegradable films using calcium-ion crosslinking and glycerol plasticization. A Box–Behnken experimental design optimized cellulose content (0.35–0.5 g), calcium chloride concentration (200–800 nm), and glycerol concentration (0.5–1.5%) to produce films with balanced mechanical and barrier properties. The optimized film was characterized for its physicochemical, mechanical, optical, antioxidant, and biodegradation properties and evaluated for fresh grape packaging. The film exhibited favorable mechanical strength of 30.82 ± 4.70 MPa, controlled water vapor permeability of 0.59 ± 0.06 10^−10^ gm^−1^ s^−1^ Pa^−1^, elongation at break of 4.36 ± 0.35%, moderate transparency of 22.95 ± 0.65% mm^−1^ at 600 nm, and ultraviolet light-blocking capability, allowing only 9.57 ± 1.44% of UV-B at 300 nm, and an IC_50_ value of 0.33, indicating moderate antioxidant potential, with 35% biodegradation after 37 days at a soil moisture of 24%. During ambient storage, grapes packaged with the film reached 15% weight loss by 13 days, while slowing changes in total soluble solids, pH, titratable acidity, total phenolic content, and vitamin C, and delaying visible quality deterioration. Compared with the uncovered control, packaged grapes maintained acceptable quality for approximately six additional days, reaching 15 days of storage. Unlike conventional polystyrene film, which promoted excessive gas accumulation and fruit cracking, the wheat straw cellulose film provided a semipermeable barrier that balanced moisture and gas exchange. The systematic optimization of these formulations, followed by comprehensive characterization of the optimized films, demonstrates the potential of wheat straw cellulose as a functional material for developing cellulose films as sustainable, biodegradable packaging materials for extending the postharvest quality of fresh produce.

## 1. Introduction

Polymeric materials have long been used by humans, but advances in polymer science during the nineteenth century and the subsequent development of synthetic plastics revolutionized modern materials manufacturing because of their exceptional versatility, durability, moldability, and resistance to chemical and physical degradation [1]. Plastic production has increased dramatically over the past century, which is projected to approach USD 1.69 trillion by 2034 [2]. The food packaging industry is driven by the desirable properties of plastics, which provide excellent mechanical strength, transparency, flexibility, barrier properties, lightweight nature, and cost-effectiveness [3]. However, most conventional plastics are derived from non-renewable petroleum resources and persist in the environment for decades after disposal. Fragmentation into microplastics and nanoplastics has emerged as a major environmental concern because these particles accumulate, raising significant risks to terrestrial and aquatic ecosystems, including human health [4,5,6]. Strategies based on the principles of reduce, reuse, and recycle (3Rs), together with restrictions on single-use plastics, have helped mitigate plastic waste, yet they cannot fully eliminate the environmental burden associated with conventional packaging materials [7]. thus, there is a need for the development of sustainable biodegradable alternatives from renewable resources.

Biobased polymers have attracted considerable attention as environmentally compatible alternatives for food packaging because of their biodegradability, renewability, favorable mechanical properties, and functional versatility. Their barrier, thermal, and optical properties further enhance their suitability for food packaging applications. Consequently, natural polymers such as cellulose, proteins, lipids, and starch, along with biodegradable synthetic polymers including polylactic acid (PLA), polyhydroxyurethanes, and polyamides, have demonstrated considerable potential as sustainable packaging materials [8,9,10]. Natural polymers such as cellulose, protein, starch, and lipids have demonstrated potential as sustainable sources of packaging due to their renewable origin, biodegradability, and functional properties, although not all biodegradable polymers are approved for food-contact applications by regulatory agencies such as the U.S. Food and Drug Administration and the European Commission [11,12]. Therefore, identifying abundant, sustainable, and economically viable biopolymer feedstocks remains essential for developing environmentally responsible packaging materials. Moreover, biodegradable packaging materials are continually engineered as functional and active systems that interact with food, the environment, and other packaging polymers to provide antioxidant, antimicrobial, ultraviolet-light-blocking, moisture-regulating, or gas-modulating functions that slow food deterioration, maintain product quality, and extend shelf life during storage [13,14]. Accordingly, developing renewable cellulose-based films that combine desirable mechanical and barrier properties with functional performance has become an important research direction for sustainable food packaging applications.

Among renewable biopolymers, cellulose is the most abundant natural polymer on Earth and is particularly attractive for biodegradable packaging because of its renewability, biodegradability, excellent mechanical properties, and film-forming capability [15,16]. The extensive intra- and intermolecular hydrogen-bonding network within cellulose provides structural integrity [17] and mechanical strength [18], making it well-suited for the fabrication of functional packaging films. Agricultural residues are an abundant yet underutilized source of cellulose that is frequently discarded or burned in open fields, contributing to greenhouse gas emissions and environmental pollution [19]. These residues can serve as a good source of low-cost, value-added packaging materials, promoting agricultural waste valorization and advancing towards a circular bioeconomy by reducing agricultural waste while also creating high-value materials.

Wheat is the second most widely cultivated cereal crop worldwide and generates substantial quantities of straw following grain harvest [20,21]. Containing approximately 30–50% cellulose, wheat straw is an inexpensive, renewable, and sustainable feedstock for producing biodegradable materials [22]. Because of its global abundance and low economic value, wheat straw is a promising lignocellulosic feedstock for developing sustainable packaging films with balanced mechanical, barrier, and functional properties. Similarly, agricultural residues such as soyhulls [23,24,25], corncobs [26,27], corn husks [28], alfalfa [29,30,31,32], grapevines [33], spent coffee grounds [34], and cow dung [35], have demonstrated considerable potential as renewable resources for producing functional packaging films and advancing the valorization of agricultural waste within a circular bioeconomy.

The performance of cellulose-based biodegradable films depends strongly on formulation variables such as cellulose concentration, crosslinking density, and plasticizer content, which directly influence mechanical strength, flexibility, moisture resistance, and barrier properties. Previous studies have commonly used calcium ions to enhance structural integrity via ionic crosslinking, promoting nanofibril formation and inducing gelation in the Zn-cellulose solution, whereas glycerol has been incorporated as a plasticizer to improve film flexibility and processability [36]. Nevertheless, the interactions among these formulation variables are highly system-dependent, making their simultaneous optimization essential for achieving balanced physicomechanical and functional properties suitable for food packaging. In our previous study, wheat straw-derived cellulose films were prepared using ZnCl_2_-mediated dissolution followed by Ca^2+^ crosslinking, and the influence of calcium chloride concentration on film properties was investigated [37]. Although that work demonstrated the feasibility of producing wheat straw-based biodegradable films, the combined effects of cellulose concentration, calcium chloride concentration, and glycerol incorporation were not systematically evaluated. Response surface methodology (RSM) provides an effective statistical framework for optimizing wheat straw cellulose films by simultaneously evaluating the individual and interactive effects of multiple formulation variables while minimizing the number of experimental trials required [38].

Beyond film optimization, the practical value of biodegradable packaging depends on its ability to preserve food quality during storage. Fresh grapes were selected as the model food system because they are one of the world’s most economically important fruit crops, with global production exceeding 80 million metric tons annually [39]. As a non-climacteric fruit, grapes are highly susceptible to postharvest quality deterioration. During storage, they rapidly undergo moisture loss, berry shriveling, rachis browning, softening, weight loss, and overall deterioration in quality, leading to reduced consumer acceptance and a shorter shelf life. Numerous postharvest technologies have been developed to reduce grape losses, including sulfur dioxide fumigation [40], irradiation [41], liquid treatments [42], modified-atmosphere packaging [43], and edible coatings [44]. Despite these advances, developing biodegradable packaging materials that effectively preserve grape quality while reducing reliance on petroleum-based plastics remains an important challenge, providing the rationale for evaluating optimized wheat straw cellulose films as sustainable packaging materials for fresh grapes.

The objective of this study was to develop functional, biodegradable packaging films from wheat straw cellulose through ZnCl_2_-mediated dissolution, calcium-ion crosslinking, and glycerol plasticization, followed by optimization of the formulation using response surface methodology (RSM). A Box–Behnken experimental design was employed to evaluate the effects of cellulose, calcium chloride, and glycerol concentrations on tensile strength, elongation at break, and water vapor permeability. The optimized films were subsequently characterized for their physical, mechanical, hydration, optical, spectroscopic, antioxidant, and biodegradation properties, and their performance in preserving fresh grapes under ambient storage conditions was evaluated. Overall, this integrated approach of optimizing the multivariate formulation demonstrates the feasibility of transforming wheat straw cellulose into functional, biodegradable packaging materials that contribute to the valorization of agricultural residues and the sustainable preservation of fresh produce.

## 2. Materials and Methods

### 2.1. Chemicals and Raw Materials

The chemicals calcium chloride, calcium sulfate, glycerol, potassium hydroxide, potassium, sodium chlorite, sulfate, and zinc chloride were purchased from VWR International, USA. Ethanol was obtained from the Chemistry Department at South Dakota State University, USA. Wheat straw and grapes were obtained from a local field in Brookings, SD, USA.

### 2.2. Methods

#### 2.2.1. Extraction of Cellulosic Residue

The wheat straw was first milled and powdered using a Glen Mills blender (Model 174937.00, Clifton, NJ, USA). The powdered material (75 g) was then treated with 1400 mL of 5% (*w*/*v*) potassium hydroxide (KOH) at 35 ± 2 °C and 300 ± 5 rpm using a digital hot plate magnetic stirrer (Thermo Scientific, Model: SP88857100, Waltham, MA, USA) for 12–14 h and washed in running water until neutralization. Then, the washed powder was treated with 1400 mL of 3% (*w*/*v*) sodium chlorite (NaClO_2_) at pH 5.0 for 6–8 h at 320 ± 5 rpm. The bleached powder was rinsed again until neutral and spread on trays or aluminum foil for drying in a hot-air oven (Cat# 151030521, Thermo Fisher Scientific, Waltham, MA, USA) set to 45 ± 2 °C for 24 h. The dried cellulose was ground further into a fine powder using a crusher (Lafati 300 A High-speed multi-functional crusher), sieved through a 60-mesh sieve to ensure uniform particle size, and stored in airtight containers. Yield of the extraction was calculated on a weight basis compared to the final weight of white dried cellulose before milling into powder. Extraction of cellulose, film formation, optimization, and characterization of the films were carried out following established protocols [31] with slight modifications.

#### 2.2.2. Optimization Process

The Box-Behnken Design (BBD) was used in Design-Expert (Trial version 13, Stat-Ease Inc., Minneapolis, MI, USA) to apply response surface methodology and fit a second-order (quadratic) model. 15 unique runs with 3 center points for the concentration of 0.3–0.5 g of cellulose, 200–800 mM CaCl_2_, and plasticizer ranging from 0.5–1.5% of cellulose were generated. The responses, tensile strength (TS), elongation at break (EB), and water vapor permeability (WVP), were evaluated.

#### 2.2.3. Film Formation

The extracted wheat cellulose (WC) was used to prepare all film compositions. WC was swollen in a 20 mL glass vial with distilled water (1.6 mL for 1 h in a water bath maintained at 83 ± 2 °C), solubilized in a 68% ZnCl_2_ solution for 30 min, crosslinked with CaCl_2_ for 10 min, and plasticized with glycerol for a further 10 min. The films were washed in a water bath and dried for 18–24 h.

### 2.3. Responses for Optimization

For the optimization process, three formulation variables—wheat straw cellulose (WC), calcium chloride (CaCl_2_), and glycerol—were selected as the independent factors, whereas tensile strength (TS), elongation at break (EB), and water vapor permeability (WVP) were selected as the response variables. The optimization aimed to maximize TS and EB while minimizing WVP. The measured responses were used to develop the response surface models.

#### 2.3.1. Tensile Strength

For TS and EB, an 8 cm long and 1 cm wide film strip was fitted into the grip of the Texture Analyzer (Stable Micro Systems, Model TA-HD plus, serial no: 5529, Surrey, UK), with a 6 cm gap maintained. The thickness of the film was measured in mm and used in the calculations. A 50 N trigger force and 15 mm/s elongation speed were employed.

The TS was calculated using Equation (1).

(1) $$TS\left(MPa\right)=\frac{Force\;used\;to\;break\;film\left(N\right)}{Surface\;area\;of\;film\left({mm}^{2}\right)}$$

#### 2.3.2. Elongation at Break

The elongation at break (EB) can also be measured simultaneously for the same strip using the same device while measuring the TS using Equation (2).

(2) $$EB\left(\%\right)=\frac{Elongation\;at\;break\left(mm\right)}{Initial\;length\left(mm\right)}\times 100$$

#### 2.3.3. Water Vapor Permeability

A glass vial was loaded with 4 g anhydrous calcium sulfate (CaSO_4_)_,_ obtaining 0.1 ± 0.1% relative humidity (RH), and the mouth of the vial (orifice of 16.4 mm diameter) was sealed with a square piece of film. The vials were then kept inside a desiccator containing a saturated potassium sulfate (K_2_SO_4_) solution (maintaining 97 ± 1% RH). The experiments were conducted at 25 ± 2 °C, and the weight was recorded every hour for 8 h. The water vapor transmission rate (WVTR) was calculated as the rate of weight change per unit time and per unit surface area of the film, and the WVP was calculated using Equation (3).

(3) $$WVP=\frac{WVTR}{P{(R}_{1}-R_{2})}\times t$$

wherein,

P: saturation vapor pressure of water (Pa) at 25 °C

R_1_: RH in the desiccator,

R_2_: RH in the cup,

t: film thickness (m).

The driving force [*P* (*R*_1_ − *R*_2_)] in the experimental condition was calculated as 3073.93 Pa.

### 2.4. Characterization

The optimized composition films were prepared, and their properties were subsequently measured. TS, EB, and WVP were measured and used to fit the model. Thickness, color, moisture content, FTIR, light transmittance, UV-blocking properties, antioxidant activity, water solubility, water absorption, water contact angle, and biodegradation.

#### 2.4.1. Color

The color of the films was measured using Nix Pro 2 color sensor (Model no: NIXPRO002, Hamilton, ON, Canada) and reported in the Hunter L*a*b* scale with a standard white plate as the background. The colors were also expressed in terms of whiteness index, yellowness index, and total color difference using Equations (4), (5), and (6), respectively,

(4) $$WI=100-[{\left(100-L\right)}^{2}+a^{2}+b^{2}{]}^{0.5}$$

(5) $$YI=142.86\frac{b_{b}}{L_{b}}$$

(6) $$\mathrm{TCD}=[{\left(L-L_{b}\right)}^{2}+{\left(a-a_{b}\right)}^{2}+{\left(b-b_{b}\right)}^{2}{]}^{0.5}$$

where,

L, a, and b: Film’s color in the Hunter scale

L_b_, a_b_, and b_b_: Background color in the Hunter scale

WI: Whiteness Index

YI: Yellowness Index

TCD: Total Color Difference

#### 2.4.2. Thickness

The film thickness was measured at five points using a Vernier caliper (RexBeti, Auburn, WA, USA).

#### 2.4.3. Moisture Content

The moisture content was determined using the gravimetric method (Equation (7)), which involved drying 2 cm × 2 cm film strips in a hot-air oven (Thermo Fisher Scientific, Waltham, MA, USA) at 110 ± 2 °C for 24 h.

(7) $$Moisture\left(\%\right)=\frac{Initial\;weight-Final\;weigth\;after\;drying}{Initial\;weight}\times 100$$

#### 2.4.4. Water Solubility

A weighed film strip of 2 cm × 2 cm, after drying in a hot-air oven (Thermo Fisher Scientific, Waltham, MA, USA) at 110 ± 2 °C for 24 h, was used for water solubility measurement. These films were immersed in 50 mL of distilled water and continuously shaken at 175 ± 5 rpm for 24 h. The samples were taken out and dried in an oven at 110 ± 2 °C for 24 h, and the final weight was recorded. The Equation (8) was used to calculate water solubility.

(8) $$Water\;solubility\left(\%\right)=\frac{Initial\;dried\;weight-Final\;dried\;weigth}{Initial\;dried\;weight}\times 100$$

#### 2.4.5. Water Absorption

The hydrophilicity of the films was assessed by tracking the water absorption of dried 2 cm × 2 cm-sized films. The film’s weight was taken after drying, and the strip’s weight was measured at 5, 15, 30, 60, 90, and 120 min by removing the strip from beaker containing 100 mL of water and dabbing off excess water from its surface. The % water absorption was calculated using Equation (9), and nine well-established models (Peleg, Singh, Peppas, Gornicki, Pilosof, Czel and Czigany, Vega-Galvez, Garcia-Pascual, and Weibull). Equations (10)–(18) were used for analyzing water absorption kinetics. The coefficient of determination (R^2^) and Root Mean Square Error (RMSE) were calculated using Equations (19) and (20) to assess the model fitness.

(9) $$Water\;absorption\left(\%\right)=\frac{Water\;absorbed\;weight-Inital\;dried\;weigth}{Initial\;dried\;weight}\times 100$$

(10) $$\frac{t}{m_{t}}=K_{2}t+K_{1}$$

(11) $${mr}_{t}=a+\frac{b\times t}{c\times t+1}$$

(12) $$\frac{{mr}_{t}/{mr}_{\infty }}{t^{1/2}}=K_{1}t^{1/2}+K_{2}$$

(13) $${mr}_{t}=a+b\times \left(1-\frac{1}{1+b\times c\times t}\right)$$

(14) $${mr}_{t}=a+\frac{b\times t}{c+t}$$

(15) $${mr}_{t}=a\times t^{m}$$

(16) $${mr}_{t}=a\times \exp\left[-\frac{b}{{\left(1-t\right)}^{\alpha }}\right]$$

(17) $${mr}_{t}=1-\exp{\left(\frac{-t}{a}\right)}^{b}$$

(18) $${mr}_{t}=m_{e}\times \left(m_{o}-m_{e}\right)\exp{\left(\frac{-t}{\beta }\right)}^{\alpha }$$

(19) R2=1−∑(yi−y^)2∑(yi−y¯)2

(20) RMSE=∑(yi−y^)2N

wherein,

m_t_: moisture content (%) at time ‘t’

mr_t_: moisture ratio at time ‘t’

mr_∞_ and m_e_: moisture ratio at 120 min

m: Slope of curve fitting in Czel and Czigany model

K_1_ and K_2_: Intercept and slope of curve fitting in Peleg model, respectively

K_2_ and K_1_: Intercept and slope of curve fitting in Peppas model, respectively

a, b, c, α, and β: constants (α = 0.9, a fixed value for the Vega-Galvez’s model only).

#### 2.4.6. Wettability

The water contact angle was measured using a 0.05 μL precise dropper from the Dropometer (Droplet Lab, Markham, ON, Canada). A sessile water drop was placed on the film surface, and its image was captured with a smartphone. The image was analyzed using the sessile drop software to calculate the water contact angle. The water contact angle was also measured at 10 s, 20 s, and 30 s to understand the changes in the film’s hydrophobicity over time. The rate of decrease in the water contact angle was fitted to a decay equation (Equation (21)).

(21) θ(t) = θ_0_·exp(−kt)

wherein,

θ(t) = Water contact angle at time (t)

θ_0_ = Initial water contact angle

#### 2.4.7. Antioxidant Activity of film

The antioxidant activity was assessed using a DPPH radical-scavenging assay. A 3 g film was cut into small pieces and mixed in 100 mL of absolute ethanol. The mixture was stirred at 100 ± 5 rpm in a glass beaker for 1 h at 25 ± 2 °C using a magnetic stirrer. Different solutions of film-to-ethanol ratios of 0.03, 0.015, 0.0075, 0.00375, 0.001875, and 0.0009375 g/mL were prepared by serial dilution. Later, 1 mL of each solution was separately loaded into a vial, and 3 mL of 0.1 mM DPPH standard solution was added, mixed vigorously, vortexed (VWR fixed-speed vortex mixer, SN# 190411003, VWR International, Radnor, PA, USA), and kept in the dark. Absolute ethanol, rather than the sample solution, was used as the control. After 30 min, the absorbance was measured at 517 nm using the UV–Vis Spectrophotometer (Model no: UV-1600PC, 10,037–436, VWR International, Radnor, PA, USA), and the radical scavenging was calculated using Equation (22). The linear regression model was fitted, and the concentration required for 50% radical scavenging was calculated and reported as the IC_50_ value of the film.

(22) $$Radical\;Scavenging\left(\%\right)=\left(1-\frac{Absorbance\;of\;sample}{Absorbance\;of\;control}\right)\times 100$$

#### 2.4.8. Light Transmittance and UV-Blocking Properties

The transmittance of electromagnetic radiation was measured using a UV–Vis spectrophotometer (Model no: UV-1600PC, 10,037–436, VWR International, Radnor, PA, USA) in the range 190 to 1060 nm at 10 nm intervals. An empty cuvette (VWR International, Radnor, PA, USA) with 100% transmittance was used as a blank. The transparency and absorption coefficient were calculated using Equations (23) and (24).

(23) $$Transparency=\frac{log(T_{600})}{t}$$

(24) $$\alpha =\frac{1}{t}\ln\left(\frac{1}{T\%}\right)$$

#### 2.4.9. FTIR

The FTIR spectra were recorded using the FTIR Spectrophotometer (PerkinElmer, Spectrum 100) in the 400–4000 cm^−1^ range and co-added from 36 scans at 4 cm^−1^ resolution. The first derivative was used to establish the peak position.

#### 2.4.10. Biodegradation

The soil was collected from a local farm for biodegradation studies, and its initial moisture content was determined to be 24 ± 2%. This value was maintained throughout the experiment by adding the required amount of water, as determined by the Pearson square method. The film strip, measuring 3 cm × 3 cm, was weighed, buried in the soil, and moisture was maintained throughout the process. The experiment was performed at 22 ± 2 °C and 47 ± 2% relative humidity. The weight was recorded every 2 days, and the % biodegradation was calculated using Equation (25).

(25) $$Biodegradation\left(\%\right)=\frac{Loss\;in\;film\;weight}{Initial\;weight}\times 100$$

Model fitness was assessed using first- and second-order kinetics (Equations (26) and (27)), as indicated by R^2^ and RMSE.

(26) Ln(y) = mx + c

(27) Ln(y) = a + bx + cx^2^

wherein,

Ln(y): natural logarithm of %biodegradation

x: biodegradation days

m and c: slope and intercept

a, b, and c: constants

### 2.5. Evaluation of Postharvest Quality 

Fresh grapes were assigned to three packaging treatments: (i) uncovered, (ii) stored in glass jars sealed with commercial polystyrene (PS) film, and (iii) stored in glass jars sealed with the optimized wheat straw cellulose biodegradable film. Samples were stored at room temperature (22 ± 2 °C) and 35 ± 3% relative humidity for 15 days. Postharvest quality attributes were evaluated at 2-day intervals, with the uncovered and PS-covered samples serving as the negative and positive controls, respectively. The evaluated parameters included percentage weight loss, total soluble solids (TSS), titratable acidity (TA), pH, ascorbic acid content, and total phenolic content (TPC), which were measured according to established methods for fresh fruits [31].

The % weight loss was measured using Equation (28).

(28) $$Weight\;loss\left(\%\right)=\frac{Initial\;weight-Weight\;at\;current\;day}{Initial\;weight}\times 100$$

Total soluble solids were measured using a handheld refractometer (0–35 °Bx), while pH was determined using a digital pH meter. Titratable acidity was determined according to AOAC Method 942.15. Briefly, 10 g of grape sample was crushed and mixed with 100 mL of distilled water. The homogenate was filtered, and a 10 mL aliquot of the filtrate was transferred to a flask containing 2–3 drops of phenolphthalein indicator. The sample was titrated with 0.1 N NaOH until a faint pink color persisted. Titratable acidity was calculated using Equation (29).

(29) $$Total\;titratable\;acidity\left(g/mL\right)=\frac{N\times 0.064\times V\times DF}{w}\times 100$$

wherein,

N: Normality of NaOH

EW: Equivalent weight of dominating acid (citric acid)

V: Volume of NaOH used as titrant to get endpoint

DF: Dilution factor

w: Sample weight

Total phenolic content (TPC) was determined according to AOAC Method 2017.13. The extraction solvent consisted of an 80:20 (*v*/*v*) ethanol–water mixture. A fresh Folin–Ciocalteu working solution was prepared by diluting the Folin reagent (1:10, *v*/*v*) with distilled water. A 1000 ppm gallic acid stock solution was prepared by dissolving 100 mg of gallic acid in 100 mL of ethanol, and standard solutions (20–100 ppm) were prepared as required for the calibration curve. Approximately 5–10 g of grape sample was homogenized in the extraction solvent and centrifuged (Model CL, SN# AD1889, International Equipment Co.,Needham Heights, MA, USA). The supernatant was collected for analysis. For the assay, 0.5 mL of extract was mixed with 2.5 mL of the Folin working solution and allowed to react for 5 min. Subsequently, 2.0 mL of 7.5% Na_2_CO_3_ solution was added, and the mixture was incubated for 30 min at room temperature. Absorbance was measured at 765 nm, and the results were expressed as mg gallic acid equivalents (GAE) per 100 g of grape sample.

Total ascorbic acid was determined according to AOAC Method 967.21. The extraction solution was prepared by dissolving 30 g of metaphosphoric acid in approximately 700 mL of distilled water, followed by the addition of 80 mL of glacial acetic acid. The final volume was adjusted to 1 L with distilled water. The standard 2,6-dichlorophenolindophenol (DCPIP) dye solution was prepared by dissolving 0.5 g DCPIP in 1 L of warm distilled water. After cooling, the solution was filtered (if necessary) and stored in an amber bottle. A fresh standard ascorbic acid solution (1 mg/mL) was prepared daily and standardized using the DCPIP solution. Approximately 10 g of grape sample was homogenized with 50 mL of the extraction solution and filtered. A 10 mL aliquot of the filtrate was titrated with the standardized DCPIP solution until a light pink color persisted for 15 s. The dye factor was calculated using Equation (30), and the ascorbic acid content was determined using Equation (31).

(30) $$Dye\;factor(F)=\frac{mg\;of\;ascorbic\;acid}{mL\;of\;dye}$$

(31) $$mg\;\%\;ascorbic\;acid=\frac{V\times F\times D}{A\times w}\times 100$$

wherein,

V: ml of dye used

D: Total extract volume

A: Aliquot volume

w: weight of sample

### 2.6. Statistical Analysis

All parameters in the study were measured in triplicate, and the average value ± standard deviation is reported. Experimental design and model fitting for tensile strength (TS), elongation at break (EB), water vapor permeability (WVP), and film optimization were carried out using Stat-Ease 360 Software (Version: 23.1.4). To compare the actual and predicted responses of the optimized film, a Welch two-sample *t*-test was conducted using RStudio 2022.07.01. Water absorption and soil biodegradation kinetics were analyzed using Microsoft Excel for Mac, Version 16.24, with the Solver Add-in.

## 3. Results and Discussion

### 3.1. Cellulose Extraction

Wheat straw and other agro-residues are of great significance as promising lignocellulosic biomass resources, with cellulose contents of 30–50%, depending on variety and growth conditions [22,45]. In this study, cellulose was extracted from wheat straw via alkali treatment followed by bleaching, yielding 38 ± 3% on a dry weight basis. This yield aligns with the reported cellulose content of wheat straw, ranging from 25.3% to 39.5% 22, indicating the effectiveness of the extraction protocol in removing non-cellulosic components such as lignin and hemicellulose. Similar yields have been reported for lignocellulosic residues from other agricultural biomasses, including around 37% from soy hulls [23], 47% from hemp [46], and 24% from alfalfa [31], where alkali and oxidative treatments were used to isolate cellulose-rich fractions. Efficient cellulose recovery is critical for film formation, as it directly affects the availability of polymer chains for dissolution, crosslinking, and network formation. The yield obtained in this study confirms that wheat straw is a viable and sustainable feedstock for producing cellulose-based biodegradable films, supporting its potential for large-scale biomass valorization.

### 3.2. Film Optimization

A Box–Behnken Design (BBD) was used to systematically optimize the formulation of wheat cellulose (WC)-based films by evaluating the combined effects of three independent variables: cellulose content (0.2–0.5 g), calcium chloride concentration (200–800 mM), and glycerol content (0.5–1.5%). These ranges were set based on preliminary trials to ensure uniform, defect-free films while staying within process feasibility limits. The BBD generated 15 experimental combinations, enabling assessment of both individual and interactive effects of the formulation variables on key performance indicators. The optimization was carried out using a multi-response approach, maximizing tensile strength (TS) and elongation at break (EB) while minimizing water vapor permeability (WVP), thereby achieving a balanced combination of mechanical integrity, flexibility, and barrier performance. This statistical approach enables the evaluation of the individual and interactive effects of formulation variables while reducing the number of experimental runs required compared with one-factor-at-a-time experimentation. Similar optimization strategies have been successfully applied to lignocellulosic film systems derived from alfalfa and soyhulls, demonstrating the effectiveness of response surface methodologies for tuning film properties [24,30]. The experimental design matrix and corresponding responses are presented in Table 1, and the results are discussed in detail in the following sections with respect to tensile strength, elongation at break, and water vapor permeability.

**Figure 1:**
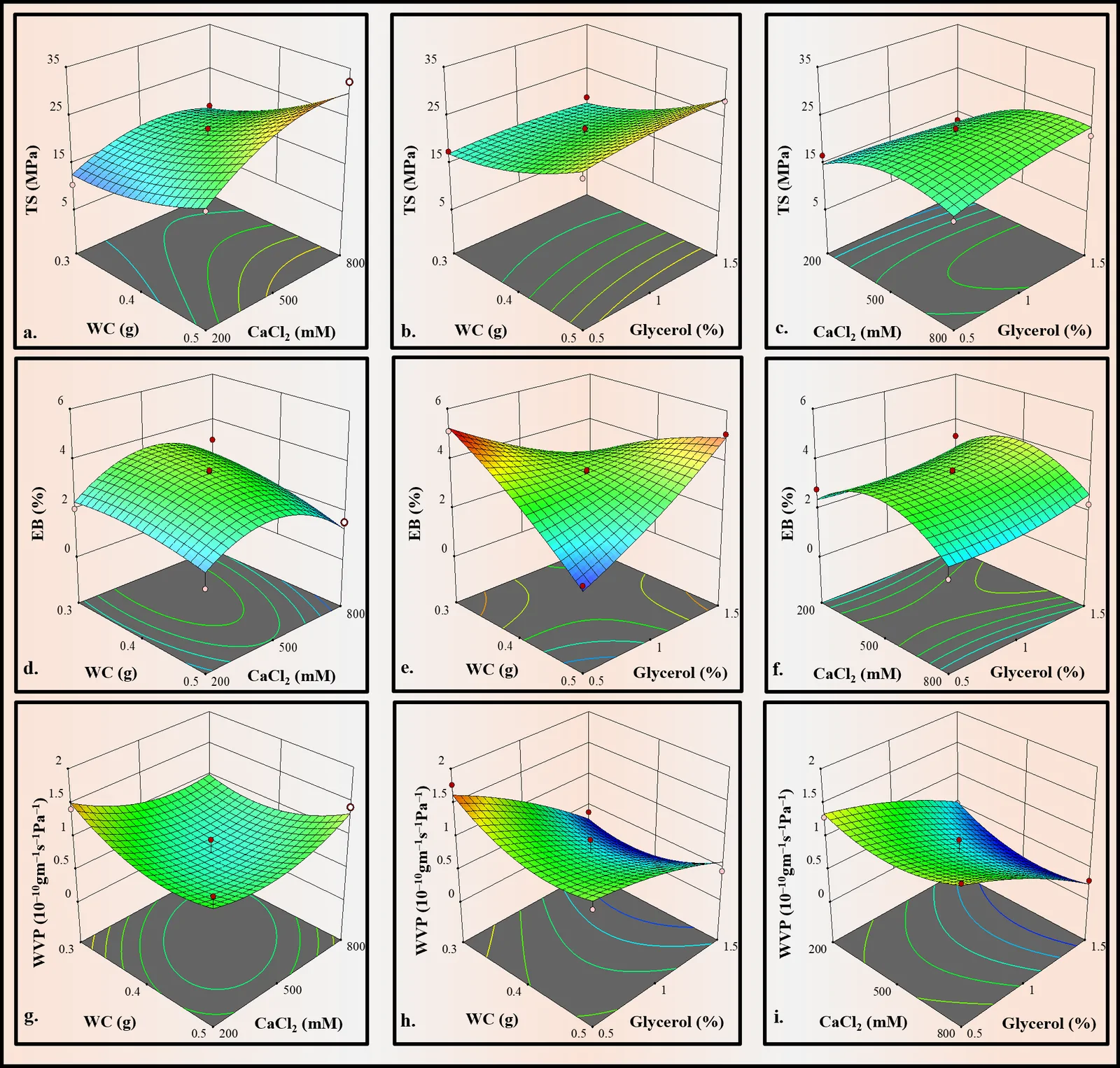
3D response surface graphs representing the effect of wheat straw cellulose (WC) and CaCl_2_, WC and glycerol, and CaCl_2_ and glycerol on the film’s (**a**–**c**) tensile strength (TS), (**d**–**f**) elongation at break (EB), and (**g**–**i**) water vapor permeability (WVP).

The relationship between input factors and their influence on the response can be identified using response surface methodology (RSM) graphs. Figure 1 helps clarify the defined experimental space and how these factors have interacted to optimize the film composition.

#### 3.2.1. Effects of Variables on Tensile Strength

The tensile strength (TS) of the wheat cellulose (WC) films ranged from 10.4 MPa for formulations with the lowest cellulose (0.3 g) and CaCl_2_ (200 mM) to 32.3 MPa for those with the highest concentrations of both components, cellulose (0.5 g) and CaCl_2_ (800 mM). This wide range highlights the strong dependence of TS on formulation variables, particularly cellulose content and calcium ion concentration. Increasing cellulose content correspondingly increased TS, as observed when comparing WC5 and WC7, and WC6 and WC8 at constant CaCl_2_ levels. This behavior can be attributed to the greater availability of polymer chains, which enhances intermolecular interactions and contributes to a more robust film network. However, the rate of increase in TS was influenced by glycerol content, indicating the interplay between structural reinforcement and plasticization. At constant cellulose levels, the effect of CaCl_2_ was particularly significant. Comparisons across runs with varying CaCl_2_ levels, WC9 and WC11, and WC10 and WC12, demonstrate that higher CaCl_2_ concentrations led to increased TS values. This trend is attributed to Ca^2+^ ions acting as crosslinking agents that promote ionic interactions between chains of cellulosic residues, resulting in a stronger, more cohesive polymer network [47].

The influence of glycerol on TS varied with CaCl_2_ concentration. At lower CaCl_2_ levels, increasing glycerol content reduced TS because of its plasticizing effect, which increases chain mobility and weakens intermolecular forces. In contrast, at higher CaCl_2_ concentrations, the effect of glycerol on TS was less pronounced, suggesting that the crosslinking effect of Ca^2+^ ions dominates over plasticization. These results suggest that both crosslinking and plasticization contribute to the observed film strength, with the relative influence depending on the formulation conditions. The combined effects of the independent variables are further illustrated by the 3D response surface plots (Figure 1a–c), which show a strong positive interaction between cellulose content and CaCl_2_ concentration. The quadratic regression model describing TS is given by Equation (32), where A, B, and C represent WC, CaCl_2_, and glycerol, respectively:

(32) $$TS=21.24+5.20A+3.47B+0.62C+1.83AB+0.62AC+1.97BC+1.82A^{2}-3.54B^{2}-0.7C^{2}$$

The statistical parameters indicate a good fit of the model, with a *p*-value of 0.0145 (<0.05), a high coefficient of determination (R^2^ = 0.9393), and a non-significant lack-of-fit (*p* = 0.1663 > 0.05), confirming the suitability of the quadratic model for predicting TS. The TS values obtained in this study are comparable to those reported for films derived from other agricultural residues, such as avocado peel (7.2–15.7 MPa) [48], banana peel (16.3–31.3 MPa) [49], and soyhulls (6.5–20.3 MPa) [23]. The TS values obtained in this study were higher than those reported in our previous work on wheat straw films [37], possibly due to differences in formulation composition and processing conditions.

Variations in TS may also stem from differences in processing conditions, including mixing, casting, and film formation methods, which influence the structural organization of the polymer network [50]. Table 2 highlights the effects of various cellulose types and their composites on film properties, including TS, EB, and WVP. Additionally, the efficiency of plasticization depends on the method of incorporation, with in situ plasticization generally yielding better matrix integration than post-treatment approaches [51]. Furthermore, intrinsic factors such as cellulose crystallinity, lignin and hemicellulose content, and the structural arrangement of cell wall components in wheat straw can also affect mechanical properties [52].

#### 3.2.2. Effects of Variables on Elongation at Break

The elongation at break (EB) of the wheat cellulose (WC) films ranged from 1.27% to 5.12%, indicating that film flexibility is strongly influenced by formulation variables, particularly the concentrations of glycerol and calcium chloride. At constant glycerol levels (runs WC1–WC4), increasing cellulose content generally decreased EB, as seen in comparisons between WC1 and WC2 and between WC3 and WC4. This behavior reflects increased rigidity of the polymer network at higher cellulose content, thereby restricting chain mobility and reducing film extensibility [64]. At constant CaCl_2_ levels (runs WC5–WC8), the relationship between cellulose content and EB was less straightforward, indicating interaction effects. For instance, while EB decreased with increasing cellulose content in some cases (WC5 vs. WC6), an opposite trend was observed in WC7 and WC8. This suggests that the effect of cellulose on flexibility depends on glycerol content, highlighting the importance of multivariable interactions.

The role of glycerol as a plasticizer was evident in runs WC9–WC12 at constant cellulose content. Increasing glycerol concentration raised EB values (WC9 vs. WC11 and WC10 vs. WC12), confirming that it enhances flexibility by occupying intermolecular spaces within the lignocellulosic matrix and reducing intermolecular forces [65]. In contrast, increasing CaCl_2_ concentration decreased EB, as observed when comparing WC9 and WC10, and WC11 and WC12 at constant glycerol levels. This reduction in elongation is attributed to enhanced ionic crosslinking, which restricts polymer chain mobility and leads to a more rigid network structure [64]. Therefore, EB is governed by the competing effects of plasticization (glycerol) and crosslinking (Ca^2+^ ions) [66].

The interactions among the independent variables are further illustrated by the 3D response surface plots (Figure 1d–f), which highlight the nonlinear and synergistic effects captured by the quadratic model. The regression equation for EB is given by Equation (33), where A, B, and C represent WC, CaCl_2_, and glycerol, respectively:

(33) $$EB=3.49-0.41A-0.10B+0.25C-0.21AB+1.64AC+0.05BC-0.27A^{2}-1.24B^{2}+0.24C^{2}$$

The statistical parameters indicate a good fit of the model, with a *p*-value of 0.0363 (< 0.05), a high coefficient of determination (R^2^ = 0.9096), and a non-significant lack-of-fit (*p* = 0.0531 > 0.05), confirming the suitability of the quadratic model for predicting EB. The EB values obtained in this study are comparable to those reported for films derived from other lignocellulosic residues, such as switchgrass (4.3–13.2%) [63], spent coffee grounds (3.8–7.9%) [34], and chia seed-based films (1.9–15.9%) [67]. However, the relatively lower EB values observed in this study, compared with some biopolymer systems, can be attributed to the strong crosslinked cellulose network, which prioritizes mechanical strength over flexibility. These observations are consistent with the view that film flexibility is influenced by the balance between crosslinking density and plasticization.

#### 3.2.3. Effects of Variables on Water Vapor Permeability

Water vapor permeability (WVP) is a critical parameter for evaluating the barrier performance of films, with lower WVP values indicating better resistance to moisture transfer and greater suitability for packaging applications. In this study, WVP values (expressed in ×10^−10^ gm^−1^ s^−1^ Pa^−1^) ranged from 0.3 to 1.77, indicating a strong dependence on formulation variables. The lowest WVP was observed for formulation WC12, which contained 0.4 g cellulose and 800 mM CaCl_2_, yielding a film with moderate tensile strength and elongation at break. In contrast, the highest WVP was recorded for WC5, which exhibited the highest elongation. This inverse relationship between elongation and barrier properties suggests that more flexible films have a more open, less densely packed structure that facilitates moisture diffusion [68].

An increase in cellulose content reduced WVP, as observed when comparing WC1 and WC2, and WC5 and WC6. This behavior can be attributed to the formation of a denser polymer matrix with increased cellulose content, which reduces free volume and limits water vapor transmission. Similarly, increasing the CaCl_2_ concentration decreased WVP (WC1 vs. WC3), indicating that ionic crosslinking enhances the network’s compactness and improves barrier properties. In contrast, glycerol markedly increased WVP. Comparisons between WC5 and WC7, and between WC6 and WC8, reveal that higher glycerol content significantly increases permeability. This is attributed to the plasticizing action of glycerol, which disrupts intermolecular interactions, increases chain mobility, and introduces free volume within the matrix, thereby facilitating water vapor diffusion [69].

The interactions among variables are further illustrated in the 3D response surface plots (Figure 1g–i), which highlight the combined effects of crosslinking and plasticization on barrier properties. The quadratic regression model for WVP is given by Equation (34), where A, B, and C represent cellulose content, CaCl_2_ concentration, and glycerol content, respectively:

(34) $$WVP=0.76-0.03A-0.07B-0.49C+0.18AB+0.19AC-0.09BC+0.26A^{2}+0.22B^{2}-0.09C^{2}$$

The statistical parameters confirm the model’s adequacy, with a *p*-value of 0.0224 (<0.05), a high coefficient of determination (R^2^ = 0.9268), and a non-significant lack-of-fit (*p* = 0.4239 > 0.05), indicating that the model reliably predicts WVP within the studied range. The WVP values obtained in this study are comparable to those reported for films derived from other biomass sources, including avocado peel (0.24–0.25; [48]), soyhull cellulose (0.7–1.9; [25]), bacterial cellulose–chitosan blends (3.65; [27]), and chitosan–silver nanoparticle films (2.18; [55]). The relatively low WVP values observed in this study indicate that the selected formulation conditions produced films with favorable barrier properties.

#### 3.2.4. Optimal Conditions and Model Validation

The optimal formulation of wheat cellulose (WC), CaCl_2_, and glycerol was determined using the developed quadratic models (Equations (28)–(30)) to maximize tensile strength (TS) and elongation at break (EB) while minimizing water vapor permeability (WVP). Statistical analysis of the response variables (Table 3) confirmed that the quadratic models adequately fit all responses, indicating their suitability for predicting optimal conditions within the studied range. The optimization identified the optimal composition as 0.5 g cellulose, 556.35 mM CaCl_2_, and 1.5% glycerol. This formulation provided a balanced combination of mechanical strength, flexibility, and barrier performance. Under these conditions, the predicted values for TS, EB, and WVP were 30.04 MPa, 4.85%, and 0.61 × 10^−10^ gm^−1^ s^−1^ Pa^−1^, respectively.

To validate the model, films were prepared using the optimized formulation (OWC film for brevity), and their properties were measured experimentally. Predicted and experimental values were compared using Welch’s *t*-test, and no statistically significant differences were observed (Table 4). This confirms the reliability and predictive capability of the developed models. The optimized formulation provided a balance among mechanical strength, flexibility, and barrier performance within the investigated formulation range. The relatively high cellulose and CaCl_2_ concentrations enhance structural integrity through increased intermolecular interactions and ionic crosslinking, while the higher glycerol content provides sufficient flexibility without significantly compromising barrier properties. These results indicate that response surface methodology was useful for identifying formulation conditions that produced desirable film properties.

### 3.3. Physical Properties of Optimized Films

#### Color and Thickness

**Figure 2:**
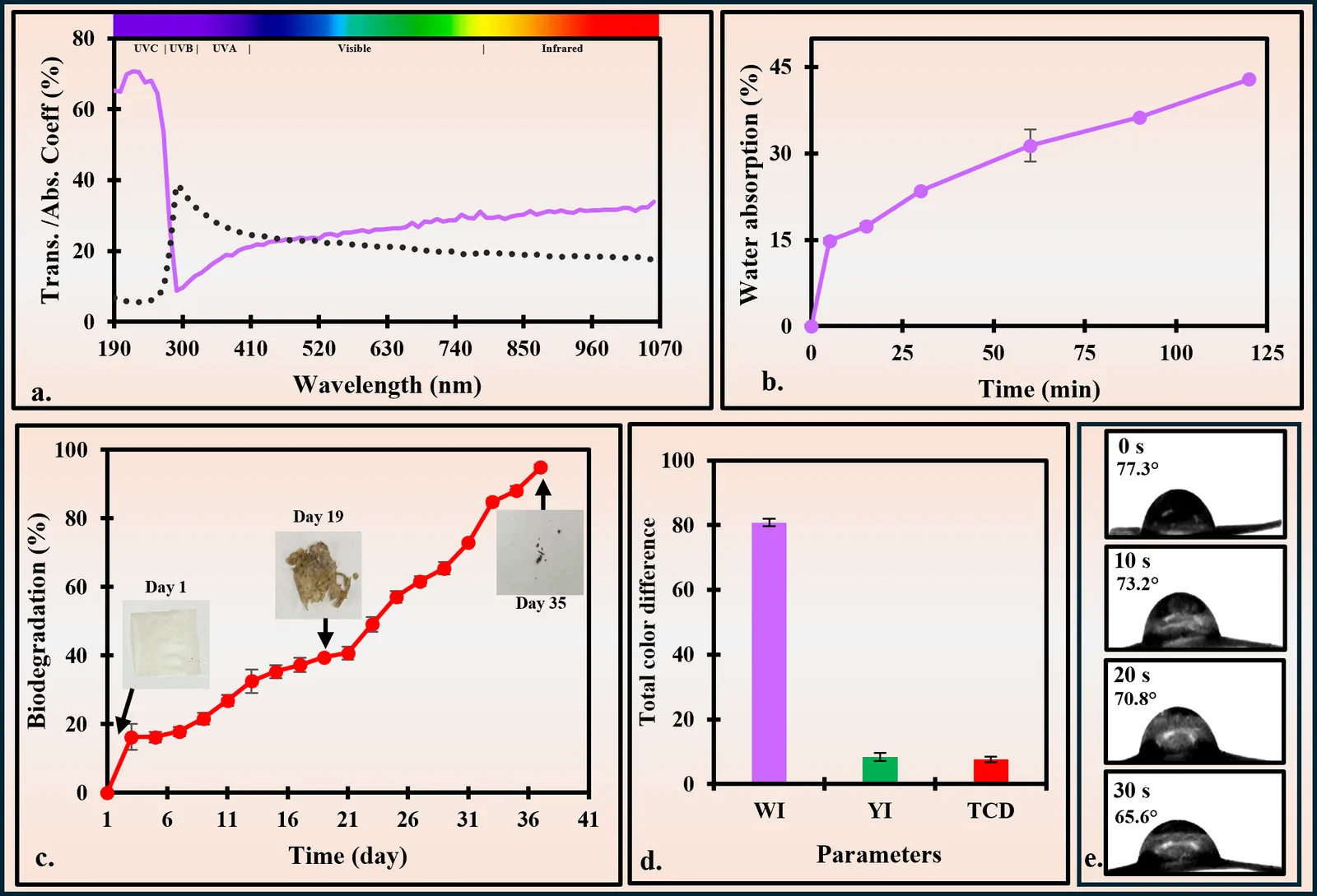
(**a**) Film’s % transmittance (purple) and absorbance (black dotted), (**b**) Water absorption trend of the film, (**c**) Biodegradation trend of the film at 24% soil moisture, (**d**) Color properties of the film Total color difference (TCD), Yellowness index (YI), and Whiteness index (WI), and (**e**) Water contact angle of the film at 10 s intervals.

The optimized wheat cellulose (OWC) films exhibited a whitish-to-slightly yellowish appearance, with L*, a*, and b* values of 81.44 ± 0.70, −1.18 ± 0.31, and 4.79 ± 1.06, respectively. The high L* value indicates a predominantly bright, light-colored film, while the negative a* and positive b* values suggest a slight greenish-yellow hue. This observation is further supported by a high whiteness index (WI) of 80.80 ± 1.19 and a relatively low yellowness index (YI) of 8.40 ± 1.28, indicating that the films maintain a desirable visual appearance suitable for packaging applications (Figure 2d). The total color difference (TCD) of 7.61 ± 0.80 ΔE indicates minimal deviation from a standard white reference, suggesting that the films possess good optical clarity. Comparable color characteristics have been reported for biodegradable films derived from soyhull cellulose and corncob cellulose, indicating that lignocellulosic films generally exhibit acceptable visual properties for practical applications [25,26].

The slight yellowness observed in the films may be attributed to light scattering within the dense polymer matrix formed via calcium-ion crosslinking, which enhances structural compactness [70]. Additionally, minor thermal or chemical modifications during processing may contribute to the formation of chromophoric groups, resulting in a slight color shift [71]. The thickness of the OWC films after drying was measured at 0.06 ± 0.009 mm across multiple locations on the film surface. The films were cast with a 1 mm clearance to ensure uniform thickness and consistent film properties [72]. The relatively low thickness observed in this study suggests efficient film formation and uniform solvent evaporation, contributing to consistent structural and functional properties.

### 3.4. Hydration Properties

#### 3.4.1. Moisture and Water Solubility

Moisture content is the amount of water retained in the film after drying and is a key parameter that influences mechanical properties such as flexibility and brittleness, as well as susceptibility to microbial growth [73]. The optimized wheat cellulose (OWC) film exhibited a moisture content of 13.63 ± 0.17%, indicating a moderate level of retained moisture. This value suggests that the films possess sufficient flexibility without excessive plasticization, while remaining relatively stable against microbial proliferation. Water solubility, on the other hand, reflects the structural integrity of the film in aqueous environments and is a critical parameter for assessing its suitability for packaging applications. The OWC films exhibited a relatively low water solubility of 21.36 ± 4.26%, indicating good resistance to dissolution compared to many other lignocellulosic films. For instance, higher solubility values have been reported for soyhull cellulose films [25] and switch grass lignocellulose [63], while the values observed in this study are comparable to those reported for wheat straw-derived films [37].

The relatively low solubility of the OWC films can be attributed to a dense cellulose network reinforced by calcium ion crosslinking, which enhances intermolecular interactions and limits water penetration. Although cellulose is inherently hydrophilic and can interact with water molecules, its crystalline structure prevents complete dissolution [74]. The presence of glycerol, while improving flexibility, may increase water affinity by introducing free volume and reducing intermolecular bonding [69]. However, in the optimized formulation, the crosslinking effect of Ca^2+^ ions appears to counterbalance the plasticizing effect, resulting in films with controlled solubility. This balance between crosslinking and plasticization aligns with trends observed in tensile strength, elongation at break, and water vapor permeability. Overall, the combination of moderate moisture content and relatively low water solubility suggests that the optimized films possess adequate structural stability for dry and low-moisture packaging applications while maintaining biodegradability under environmental conditions.

#### 3.4.2. Water Absorption Kinetics

Water absorption (WA) behavior provides important insights into the time-dependent interaction of films with moisture and is critical for understanding film porosity, structural integrity, and stability under humid or wet conditions. The optimized wheat cellulose (OWC) film exhibited a water absorption capacity of 42.89 ± 0.14% after 120 min of immersion under continuous stirring. The absorption profile showed rapid initial uptake followed by a gradual approach to equilibrium and the increase in water absorption % was noted at 5, 15, 30, 60, 90, 120 min from 14.80 ± 0.52%, 17.31 ± 0.52%, 23.51 ± 0.41%, 31.35 ± 2.80%, 36.27 ± 0.25%, and 42.89 ± 0.14%, respectively. This behavior indicates rapid surface sorption followed by slower diffusion of water into the film’s internal structure.

To better understand the absorption mechanism, the experimental data were fitted to seven kinetic models, including Peleg, Singh, Peppas, Gornicki, Pilosof, Czel and Czigany, and Vega-Galvez. Model performance was evaluated using the coefficient of determination (R^2^) and root mean square error (RMSE). Although all models captured the general trend, the Pilosof model provided the best fit, with the highest R^2^ (0.9993) and the lowest RMSE (0.0063), indicating excellent agreement with the experimental data. The parameters of the Pilosof model further elucidate the absorption mechanism. The ‘*a*’ value (0.126) reflects rapid initial water uptake, corresponding to surface adsorption. The sum of parameters ‘*a*’ and ‘*b*’ (0.936) represents the asymptotic maximum absorption capacity on a fractional basis, suggesting a high equilibrium moisture uptake potential. The parameter ‘*c*’ (206.02 min) corresponds to the characteristic time required to approach equilibrium, indicating a relatively gradual saturation process. These trends are consistent with the absorption curve (Figure 2b), which shows an initial steep increase followed by a plateau. The rate constants of the Peleg and Pilosof models are related, and the Pilosof model is more closely associated with the time to absorb half the maximum amount of water; the Pilosof model seems to fit better as the rate of water absorption slows after a certain time [75].

The observed water absorption behavior can be attributed to cellulose’s hydrophilic nature and the film’s structural characteristics. While glycerol enhances water affinity by increasing free volume and chain mobility, Ca^2+^-induced crosslinking restricts excessive swelling by maintaining structural integrity. This balance yields controlled water uptake, preventing rapid disintegration while still allowing interaction with moisture [47,76]. Overall, the kinetic analysis suggests that water uptake involves both rapid surface sorption and subsequent diffusion into the film matrix, which is desirable for applications where short-term moisture exposure is expected without immediate structural failure. This controlled absorption behavior aligns with the moderate wettability and low solubility observed for the optimized films.

#### 3.4.3. Water Contact Angle

Water contact angle (WCA) analysis provides insight into the surface wettability of films, distinguishing between hydrophilic (θ_0_ < 90°) and hydrophobic (θ_0_ > 90°) behavior. The optimized wheat cellulose (OWC) film exhibited an initial contact angle (θ_0_) of 77.3 ± 1.9°, confirming its hydrophilic nature. Over 30 s, the contact angle gradually decreased to 65.6 ± 1.8°, indicating progressive spreading of the water droplet on the film surface, as shown in Figure 2e. This time-dependent decrease reflects the film’s dynamic wetting behavior, governed by both surface energy and liquid penetration into the film matrix [77]. The hydrophilic nature of the OWC films can be attributed to hydroxyl groups in cellulose, which facilitate hydrogen bonding with water molecules. Additionally, incorporating glycerol as a plasticizer further enhances surface wettability by increasing the availability of polar functional groups and promoting water affinity.

The wetting rate constant (*k*) for the OWC film was determined to be 0.005 s^−1^, indicating moderate wetting kinetics. According to established classifications, higher *k* values (>0.01) are associated with rapid wetting and highly hydrophilic surfaces, whereas lower values (<0.002) correspond to slower wetting and more hydrophobic behavior [78]. The intermediate *k* value observed in this study suggests controlled water spreading on the film surface, which may help maintain structural integrity during short-term moisture exposure. The observed wettability reflects the balance between hydrophilicity introduced by cellulose and glycerol, and the structural stabilization provided by Ca^2+^ crosslinking. While the film surface readily interacts with water, the underlying crosslinked network limits excessive swelling and dissolution, consistent with trends observed in water absorption and solubility studies.

### 3.5. Antioxidant Activity

Antioxidant activity (AA) is an important functional property of biodegradable films, particularly for packaging applications, as it can help retard oxidative degradation and extend the shelf life of packaged products. In addition to acting as a physical barrier, films with inherent antioxidant properties can provide radical-scavenging and metal-chelating functions, thereby enhancing overall preservation performance. The optimized wheat cellulose (OWC) film exhibited radical-scavenging activity (RSA) of 10.3%, with an IC_50_ of 0.33, indicating moderate antioxidant potential. The observed RSA is higher than that reported for alfalfa cellulose films of 7.32% 31, and soyhull lignocellulosic residue films of 5.5% 25, but lower than that of films derived from spent coffee grounds of 26.1% [34], which are known to contain higher levels of phenolic compounds.

The antioxidant activity observed in the OWC films can be attributed to multiple factors. Cellulose contributes weak radical-scavenging activity through its hydroxyl groups, as reported for cellulose derivatives such as cellulose acetate [79]. However, the primary contribution likely comes from residual phenolic compounds associated with wheat straw biomass. Wheat straw contains phenolic acids and aldehydes, including p-hydroxybenzoic acid, vanillic acid, ferulic acid, cinnamic acid, and p-coumaric acid, which are predominantly associated with the lignin fraction [80,81]. During extraction, small amounts of these phenolic compounds may remain bound to the cellulose matrix or be co-extracted due to linkages between lignin and hemicellulose components, such as ferulate-mediated crosslinks in arabinoxylans [82]. These residual compounds can contribute to the observed antioxidant activity of the films. The observed antioxidant activity may be advantageous in applications where a degree of radical-scavenging capacity is desirable. Such films can help mitigate oxidative reactions, including lipid oxidation and enzymatic browning, and may also provide limited antimicrobial benefits.

### 3.6. Spectroscopic Analysis

#### Transparency, Transmittance, and Absorption Coefficients

**Figure 3:**
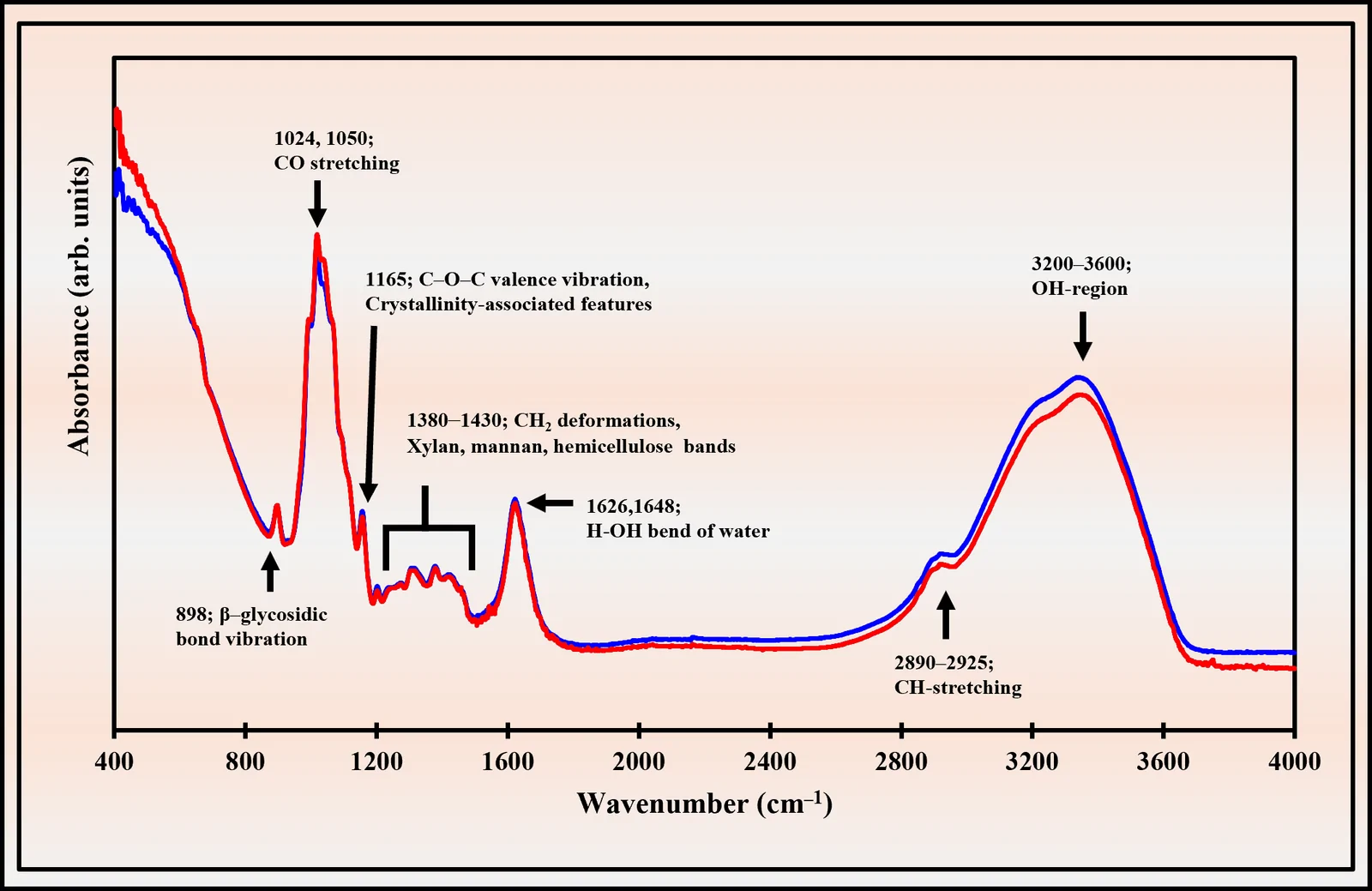
FTIR spectra of wheat straw cellulosic residue (WC) and optimized wheat cellulose (OWC) film showing peak shifts and variations during film making.

Transparency is an important optical property of films, affecting product visibility and protection against light-induced degradation. It reflects a material’s ability to transmit image-forming light. The optimized wheat cellulose (OWC) film exhibited a transparency of 22.95 ± 0.65% mm^−1^ at 600 nm, indicating moderate clarity suitable for packaging applications where partial visibility is desirable. Film transparency is influenced by several factors, including thickness, polymer–plasticizer interactions, crystallinity, and the presence of chromophoric groups. In the present study, the observed transparency may be attributed to the combined effects of glycerol-induced plasticization, calcium-ion crosslinking, and the structural organization of the cellulose matrix, with minor contributions from thermal processing and possible chromophore formation [83,84,85,86,87,88]. The OWC films exhibited wavelength-dependent transmittance across different spectral regions, with values ranging from 8.71% to 70.7%. In the UV-C region (250 nm), the films showed relatively high transmittance (68.1 ± 0.87%), whereas in the UV-B region (300 nm), transmittance decreased to 9.57 ± 1.44%. In the UV-A region (350 nm), transmittance increased slightly to 16.7 ± 2.01%. In the visible region (390–700 nm), transmittance ranged from 20.1 ± 2.45% to 28.1 ± 1.50%, indicating moderate light transmission sufficient to maintain product visibility. Transmittance increased further toward the infrared (IR) region, as seen in Figure 3.

The absorption coefficient provides an intrinsic measure of light attenuation independent of film thickness. The OWC films exhibited the highest absorption in the UV-B region, with values reaching 34.5 ± 2.05, compared with lower values in the UV-C region (6.8–9.9) and decreasing absorption in the visible and IR regions. The high UV-B absorption indicates effective blocking of harmful UV radiation, which is advantageous for protecting light-sensitive products from photo-induced degradation. The optical properties suggest that the films may provide both product visibility and partial protection against UV radiation. Similar trends have been reported for biodegradable films derived from soyhull and corncob cellulose, where selective light attenuation enhances their suitability for packaging applications [23,26].

### 3.7. Fourier Transmission Infrared (FTIR) Spectroscopic Determination

The FTIR determination of cellulose and the optimized film was performed, and studies of changes in the peaks and in the arrangement characteristics of cellulose’s structural components were conducted; major peaks are shown in Figure 3.

The FTIR spectra of WC (wheat cellulose) and OWC (optimized wheat cellulose) films closely exhibit characteristic absorption bands consistent with cellulose, confirming a cellulose-rich matrix. The broad absorption band at 3340–3400 cm^−1^, 2890–2925 cm^−1^, and 1630–1650 cm^−1^ (Figure 3) corresponds to O–H stretching vibrations associated with extensive intra- and intermolecular hydrogen bonding, C–H stretching of the cellulose backbone, and H–O–H bending of absorbed water, which is consistent with the hydrophilic nature of the cellulose structure. The absence of a prominent band near 1735 cm^−1^ further supports the removal of hemicellulose and other non-cellulosic components during alkali treatment and bleaching.

Compared to WC, OWC retained principal cellulose bands, with slight changes in O–H regions. Slight shifts and changes in peak intensity were observed around 1024 cm^−1^ and within the 1000–1200 cm^−1^ range, associated with C–O and C–O–C stretching vibrations. A prominent peak at approximately 898 cm^−1^, corresponding to C_1_–H deformation and β-glycosidic linkages, was observed in both WC and OWC films, confirming the integrity of the cellulose backbone. These variations suggest subtle changes in the structural organization of the cellulose chains, potentially indicating partial transformation to regenerated cellulose (cellulose II) during ZnCl_2_ dissolution and subsequent Ca^2+^ crosslinking. No new peaks associated with chemical modification were observed, indicating that the fundamental cellulose structure remained unchanged. Film formation primarily involved physical and intermolecular interactions, and detailed peak assignments are presented in Table 5.

### 3.8. Soil Biodegradation

The biodegradation behavior of the optimized wheat cellulose (OWC) film showed a gradual, nonlinear increase over time, indicating progressive breakdown under soil conditions. Biodegradation involves the loss of physicomechanical integrity, fragmentation of the material, and chemical modification driven by microbial activity, enzymatic action, and environmental factors such as moisture, temperature, and oxygen availability [94]. In this study, biodegradation experiments were conducted under controlled laboratory conditions, with soil moisture regularly monitored and maintained to ensure consistency. A rapid increase in degradation was observed between days 7 and 23, during which approximately 50% of the film’s weight was lost. Continued degradation led to more than 90% disintegration by day 37. The biodegradation progression at specific time intervals was recorded as 21.66% (day 9), 39.47% (day 19), 65.42% (day 29), and 94.89% (day 37), demonstrating efficient film breakdown within a relatively short period (Figure 2c).

The high biodegradability of the OWC film is attributed to its hydrophilic nature and functional groups that facilitate microbial interactions. The abundance of hydroxyl (–OH) groups in cellulose and glycerol enhances water uptake, promoting swelling and making the film more accessible to microbial enzymes. This increased moisture interaction accelerates hydrolytic and enzymatic degradation [84]. Additionally, the biodegradability of cellulose, combined with partial structural loosening from plasticization, promotes rapid disintegration in soil. Biodegradation kinetics were analyzed using first- and second-order models, with the second-order model providing the best fit (R^2^ = 0.9887, RMSE = 0.06). This suggests that degradation is influenced by both the concentration of degradable material and environmental interactions, reflecting a complex degradation mechanism. The observed biodegradation behavior aligns with reports on other lignocellulosic films, including those derived from banana peel fiber, avocado peel, soyhull residue, and alfalfa biomass [24, 31, 48, 49, 95]. The substantial biodegradation observed under the tested conditions demonstrates the biodegradable nature of films and supports their potential use in applications where environmental degradability is desired.

### 3.9. Evaluation of Postharvest Quality of Grapes

The effectiveness of the optimized wheat cellulose (OWC) films for postharvest preservation was evaluated by monitoring changes in the physicochemical and nutritional quality of grapes over 15 days of storage at room temperature. Quality deterioration was assessed through visual observations and measurements of weight loss, total soluble solids (TSS), pH, titratable acidity, vitamin C, and total phenolic content (TPC).

Visual quality served as the primary indicator of postharvest deterioration because no single biochemical threshold defines acceptable grape freshness. Instead, quality loss was evaluated based on visible shriveling, berry cracking, leakage, and mold development, as well as changes in measured physicochemical and nutritional attributes. Greater weight loss reduced titratable acidity, increased pH and TSS, and decreased vitamin C and phenolic contents were considered indicators of progressive fruit deterioration. All treatments exhibited some degree of moisture loss and berry shriveling during storage, although mold growth was not observed. Gas accumulation beneath both the polystyrene (PS) and optimized wheat cellulose (OWC) films became apparent on Days 5 and 7, causing the films to bulge slightly. By Day 9, several berries in the PS-covered jars had cracked, likely because the relatively low gas permeability of the PS film allowed internal pressure to build in the package headspace. The elevated internal pressure, together with enzymatic softening of the fruit tissues during storage, may have weakened the berry skins and promoted cracking. After juice leaked from the damaged berries, visible mold growth developed on Day 11.

In contrast, berries packaged with the OWC film remained intact throughout storage. The semipermeable nature of the biodegradable film likely allowed a more balanced exchange of O_2_ and CO_2_, preventing excessive pressure buildup while maintaining a favorable modified atmosphere around the fruit. This controlled gaseous environment may also have delayed fungal development and preserved overall fruit quality during storage.

#### 3.9.1. Weight Loss

**Figure 4:**
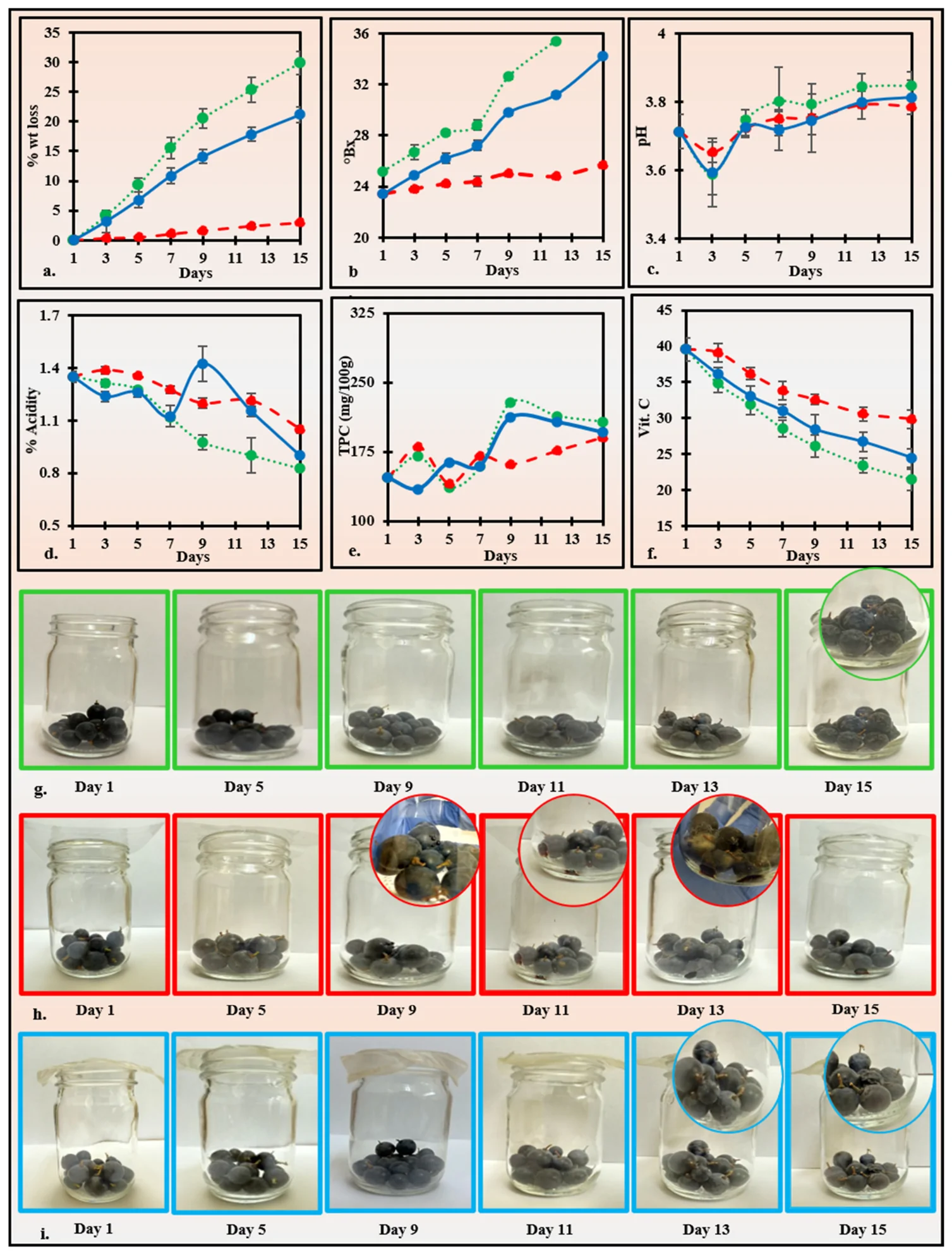
(**a**) % weight loss of grapes, (**b**) Total soluble solids (TSS), (**c**) pH, (**d**) % acidity, (**e**) Total phenolic content (TPC), (**f**) ascorbic acid content, and (**g**–**i**) Changes in grapes during storage of 15 days; uncovered jar (green), PS covered jar (red), and OWC film covered jar (blue).

Fresh grapes remain physiologically active after harvest and continuously lose water through transpiration and respiration, making them highly susceptible to postharvest weight loss and quality deterioration [96]. As expected, grapes stored in uncovered jars (UJ) showed the greatest weight loss because unrestricted exposure to the surrounding environment accelerated moisture loss. This rapid dehydration reduced berry firmness and turgor, resulting in visible shriveling by Day 9, when weight loss approached 20% (Figure 4a).

In contrast, grapes packaged in jars sealed with commercial polystyrene film (PSJ) exhibited the lowest weight loss, only 3% after 15 days, confirming the excellent moisture-barrier properties of PS film. Grapes packaged with the optimized wheat cellulose film (OWFJ) showed an intermediate weight loss of approximately 21% by Day 15, indicating that the biodegradable film effectively reduced moisture loss compared with the uncovered control while allowing greater moisture exchange than the PS film. This behavior is consistent with the semipermeable nature of cellulose-based films, which moderate water vapor transfer rather than completely restricting it.

Although the OWFJ grapes experienced greater weight loss than the PSJ treatment, the biodegradable film slowed moisture loss and maintained fruit quality throughout storage. At Day 15, the berries showed visible shriveling and reduced market appeal; however, previous studies have reported that grape skin wrinkling may become apparent once weight loss exceeds approximately 5% [96]. The higher threshold observed in the present study may reflect differences in grape cultivar, storage conditions, packaging configuration, or environmental humidity.

#### 3.9.2. Total Soluble Solids (TSS)

Total soluble solids (TSS) increased across all treatments during storage, although the magnitude of the change varied considerably by packaging system (Figure 4b). The increase in TSS is primarily attributed to moisture loss, which concentrates soluble constituents within the fruit, as well as the continued breakdown of structural carbohydrates and other macromolecules during postharvest metabolism [97]. Similar increases in TSS during storage have been reported for strawberries [23] and grapes [97,98].

The uncovered grapes (UJ) exhibited the greatest increase in TSS, rising from 25.2 ± 0.2 to 35.4 ± 0 °Bx within 12 days, reflecting rapid moisture loss and accelerated physiological changes. In contrast, grapes packaged with commercial polystyrene film (PSJ) showed only a slight increase from 23.4 ± 0.2 to 25.7 ± 0.2 °Bx after 15 days, indicating superior moisture retention and greater compositional stability. Grapes packaged with the optimized wheat cellulose film (OWFJ) exhibited an intermediate response, with TSS increasing from 23.4 ± 0 to 34.2 ± 0 °Bx after 15 days. This moderate increase suggests that the semipermeable biodegradable film partially reduced moisture loss while allowing sufficient gas exchange to support normal physiological processes during storage. Although the final TSS values of UJ and OWFJ grapes were comparable, the increase in TSS was more gradual in the OWFJ treatment, indicating that the biodegradable film delayed moisture loss and postharvest compositional changes.

Overall, the TSS results demonstrate that the permeability of the packaging material strongly influenced moisture retention and the accumulation of soluble solids. The optimized wheat cellulose film provided an intermediate preservation effect, reducing compositional changes relative to the uncovered control while maintaining a more balanced storage environment than the low-permeability PS film.

#### 3.9.3. pH and Acidity

Changes in pH and titratable acidity during storage were relatively small and were not significantly different among the treatments (Figure 4c,d). Nevertheless, a gradual increase in pH accompanied by a corresponding decrease in titratable acidity was observed in all samples, indicating the progressive depletion of organic acids during postharvest storage.

The uncovered grapes (UJ) exhibited the greatest increase in pH, rising from 3.71 to 3.85, whereas grapes packaged with the optimized wheat cellulose film (OWFJ) and commercial polystyrene film (PSJ) showed smaller increases to 3.81 and 3.79, respectively. The more pronounced pH increase in the uncovered treatment is consistent with accelerated moisture loss, continued respiration, and increased metabolic activity, all of which contribute to the depletion of organic acids during storage.

A similar trend was observed for titratable acidity. The uncovered grapes exhibited the greatest reduction in acidity, while the PSJ treatment maintained the highest acidity throughout storage. Grapes packaged with the optimized wheat cellulose film showed an intermediate decrease in titratable acidity, from 1.35 ± 0.03 to 0.90 ± 0.02% (as tartaric acid). This intermediate response indicates that the semipermeable biodegradable film partially slowed the loss of organic acids by reducing moisture loss and moderating the storage atmosphere, although its preservation effect was less pronounced than that of the low-permeability PS film.

Overall, the inverse relationship between pH and titratable acidity reflects the gradual loss of freshness during storage [99]. Among the three treatments, the uncovered grapes deteriorated most rapidly, whereas the PSJ treatment best preserved physicochemical stability. The optimized wheat cellulose film provided an intermediate level of preservation, demonstrating its ability to delay compositional changes while maintaining a more balanced storage environment than the uncovered control.

#### 3.9.4. TPC

Phenolic compounds are important secondary metabolites that contribute to fruit quality, including color, taste, and flavor, while also providing antioxidant activity and potential health benefits. Changes in total phenolic content (TPC) during postharvest storage have been widely reported; however, the direction and magnitude of these changes vary depending on grape cultivar, storage conditions, packaging system, postharvest treatments, and the metabolic activities occurring during storage [99].

As shown in Figure 4e, the TPC of grapes in all treatments exhibited a slight initial decline followed by an increase after Day 7. This trend may be attributed to the gradual release of phenolic compounds from cellular structures as fruit tissues softened during storage, increasing their extractability [100,101]. In addition, because TPC is expressed on a fresh-weight basis, moisture loss during storage may have concentrated phenolic compounds, resulting in higher measured values, particularly at later storage stages. Although all treatments showed a similar overall trend, differences in the extent of moisture loss and physiological changes likely influenced the measured TPC. The packaging systems moderated these changes by affecting water loss and the storage atmosphere, thereby influencing phenolic metabolism throughout storage. Similar increases in TPC during postharvest storage have been reported for grapes under comparable storage conditions [100,101].

#### 3.9.5. Vitamin C

Vitamin C (ascorbic acid) content declined in all treatments during storage, indicating progressive deterioration of the nutritional quality of the grapes (Figure 4f). The retention of ascorbic acid during postharvest storage is influenced by several factors, including oxygen availability, oxidative stress, respiration, temperature, pH, and storage duration [102]. Prolonged storage accelerates the oxidation of ascorbic acid to dehydroascorbic acid, which is subsequently degraded, resulting in a gradual loss of vitamin C.

The uncovered grapes (UJ) exhibited the greatest reduction in vitamin C, decreasing from 39.6 ± 1.6 to 21.4 ± 1.5 mg% ascorbic acid. This substantial loss is attributed to unrestricted exposure to oxygen, which accelerates oxidative degradation and increases respiratory activity during storage. In contrast, grapes packaged with commercial polystyrene film (PSJ) retained the highest vitamin C content (29.86 ± 1.3 mg% ascorbic acid), reflecting the superior moisture and gas barrier properties of the PS film, thereby reducing oxidative stress and slowing nutrient degradation.

Grapes packaged with the optimized wheat cellulose film (OWFJ) retained an intermediate vitamin C content of 24.48 ± 1.3 mg% ascorbic acid, demonstrating partial protection against nutrient loss. Although reduced oxygen availability generally slows oxidative degradation, excessive restriction of gas exchange may also contribute to ascorbic acid degradation through alternative metabolic pathways under low-oxygen conditions [103]. The intermediate retention observed in the OWFJ treatment suggests that the semipermeable biodegradable film moderated oxygen transfer sufficiently to delay vitamin C degradation while maintaining a favorable storage atmosphere.

Overall, the vitamin C results demonstrate that packaging significantly influenced the preservation of nutritional quality during storage. The PSJ treatment provided the greatest protection against ascorbic acid loss, whereas the optimized wheat cellulose film effectively delayed vitamin C degradation compared with the uncovered control. These findings agree with previous studies showing that packaging systems that reduce oxygen exposure and physiological stress better preserve ascorbic acid during postharvest storage [102,103]. The summary of results for the optimized film is listed in Table 6.

## 4. Conclusions

Cellulose extracted from wheat straw was successfully converted into biodegradable packaging films through ZnCl_2_-mediated dissolution, calcium-ion crosslinking, and glycerol plasticization. Response surface methodology, based on a Box–Behnken experimental design, enabled systematic optimization of the formulation, identifying an optimal composition of 0.5 g cellulose, 556.35 mM CaCl_2_, and 1.5% glycerol. The optimized films exhibited a balanced combination of mechanical strength (30.04 MPa), elongation at break (4.85%), low water vapor permeability (0.61 × 10^−10^ g m^−1^ s^−1^ Pa^−1^), moderate transparency, UV-light attenuation, controlled water uptake, antioxidant activity, and rapid biodegradation, reaching approximately 95% degradation within 37 days under soil conditions. Furthermore, the optimized films effectively preserved the postharvest quality of fresh grapes during ambient storage, demonstrating their practical applicability as sustainable food packaging materials. Overall, this study demonstrates the feasibility of transforming wheat straw, an abundant agricultural residue, into high-value biodegradable packaging materials, thereby advancing agricultural waste valorization and the development of sustainable food packaging technologies.

The application of optimized wheat cellulose (OWC) films for grape packaging further demonstrated their potential for postharvest preservation. A limitation of this study was the evaluation of a single grape variety, selected because it was seasonally available and had not undergone commercial postharvest preservation treatments that could confound the evaluation of film performance. Consequently, the findings should be interpreted in the context of the performance of the optimized film rather than as representative of all grape varieties. Compared with the uncovered control, the OWC films reduced moisture loss during early storage, prevented berry cracking and subsequent mold development by enabling balanced gas exchange within the package headspace, and maintained postharvest quality throughout storage. Although commercial PS film provided superior moisture retention, the OWC films offered an effective biodegradable alternative that balanced moisture control with controlled gas permeability, thereby preserving fruit integrity while eliminating the need for petroleum-based packaging materials.

Taken together, these findings demonstrate that wheat straw, an abundant agricultural residue, can be converted into functional, biodegradable packaging materials suitable for preserving fresh produce. Integrating formulation optimization with postharvest performance evaluation provides a practical framework for designing cellulose-based packaging with tailored properties for specific food applications. These findings advance the value-added utilization of agricultural residues and support the development of sustainable food packaging technologies within a circular bioeconomy.

## Figures and Tables

**Table 1:** Independent variables are wheat straw cellulose (WC) in g, CaCl_2_ in mM, and Glycerol in %, while the response variables are tensile strength (TS) in MPa, elongation at break (EB) in %, and water vapor permeability (WVP) in 10^−10^ gm^−1^ s^−1^ Pa^−1^.

Run	WC	CaCl_2_	Glycerol	TS	EB	WVP
WC1	0.3	200	1	10.40 ± 0.6	2.00 ± 0.3	1.42 ± 0.09
WC2	0.5	200	1	18.87 ± 1.2	1.27 ± 0.4	1.25 ± 0.08
WC3	0.3	800	1	16.52 ± 0.8	3.10 ± 0.4	0.84 ± 0.06
WC4	0.5	800	1	32.30 ± 1.3	1.56 ± 0.2	1.42 ± 0.30
WC5	0.3	500	0.5	17.59 ± 0.5	5.12 ± 0.4	1.77 ± 0.60
WC6	0.5	500	0.5	25.03 ± 0.8	1.37 ± 0.3	1.07 ± 0.40
WC7	0.3	500	1.5	18.47 ± 0.6	2.29 ± 0.6	0.38 ± 0.07
WC8	0.5	500	1.5	23.38 ± 1.1	5.08 ± 0.5	0.45 ± 0.10
WC9	0.4	200	0.5	16.74 ± 1.2	2.80 ± 0.4	1.30 ± 0.30
WC10	0.4	800	0.5	16.93 ± 1.4	1.62 ± 0.4	1.42 ± 0.30
WC11	0.4	200	1.5	13.15 ± 0.7	3.28 ± 0.2	0.53 ± 0.03
WC12	0.4	800	1.5	21.21 ± 0.7	2.29 ± 0.5	0.30 ± 0.03
WC13	0.4	500	1	22.60 ± 0.9	3.57 ± 0.3	0.95 ± 0.04
WC14	0.4	500	1	20.93 ± 0.4	3.62 ± 0.3	0.59 ± 0.02
WC15	0.4	500	1	20.20 ± 0.6	3.28 ± 0.6	0.73 ± 0.08

**Table 2:** Comparison of tensile strength (TS in MPa), elongation at break (EB in %), water vapor permeability (WVP in 10^−10^ gm^−1^ s^−1^ Pa^−1^), water solubility (WS in %), antioxidant activity (RSA in %), and 90% film biodegradation (BD in days) of films from some biopolymers and their composites.

Biopolymer	TS	EB	WVP	WS	RSA	BD	Reference
Wheat straw	10.4–32.3	1.27–5.12	3–17.7	17.1–25.6	10.3	37	This study
Alfalfa	16.9	10.1	0.47	9.2		17	[31]
Avocado peel fiber	7.2–15.7	15.7–21.8	2.4–2.5	15.7–21.8	-	28	[48]
Alginate	0.7–2.1	41.9–54.2	16.68–25.02	-	4.57–23.09	-	[53]
Alginate, cellulose nanofibers	22–31	-	-	1.7–8.6	-	35	[54]
Bacterial cellulose, chitosan nanocomposites	21.1–42.9	22.5–33.8	2.02–3.65	16.5–22.4	-	-	[55]
Banana peel	16.3–31.3	4.9–13	2.4–3.6	24.5–65.7	-	30	[49]
Carboxymethyl cellulose	6.7–45.1	2.1–22	48.6–214	-	-	-	[56]
Cellulose protein composites	16.2–31.3	10.7–12.7	0.9–1.2	26.5–69.6	-	19–25	[57]
Cellulose with lignin nanoparticles	110.4	-	0.11	36.6	-	12	[58]
Corncob cellulose	4.7	15.4	1.8	64.1	15.1	29	[26]
Chitosan-cellulose nanocomposite	17.4–33.8	2.4–4.7	0.12	-	-	-	[59]
Chitosan-protein-cellulose	6.41	97.46	-	-	-	-	[60]
Chitosan-soyhull cellulose	6.3–15.9	9.4–30.2	0.64–0.9	34.2–69.5	-	25–39	[61]
Cow dung cellulose	2.2–4.2	9.7–11	1.0–1.5	51.7–67.9	-	21	[35]
Grapevine cellulose	15.4–18.2	6.1–8.6	0.7–1.4	13.9–18.6	-	15–17	[33]
Oat straw	4.2–17.2	5.8–8.3	0.5–0.7	43.9–68.5	-	-	[62]
Spent coffee grounds lignocellulose	8.4–26.8	3.8–7.9	0.8–1.8	-	-	45	[34]
Switch grass lignocellulose	9.9–14.7	3.4–4.7	0.1–0.2	58.7–71.2	-	35	[63]

**Table 3:** ANOVA of quadratic model fitting for the responses Tensile strength (TS), Elongation at break (EB), and water vapor permeability (WVP) of WC films. The model’s *p*-value and lack-of-fit value were used as indicators to assess the model’s fit.

Response	Source	F-Value	*p*-Value	R^2^
TS	Model Lack of fit	8.60 5.17	0.0145 0.1663	0.9393
EB	Model Lack of fit	5.59 18	0.0363 0.0531	0.9096
WVP	Model Lack of fit	7.03 1.50	0.0224 0.4239	0.9268

**Table 4:** Summary of statistics confirming that the predicted optimized values match the experimental results for the film made with the optimized composition.

Parameter	Predicted Value	Obtained Result	*p*-Value	Significance (α = 0.05)
TS	30.00 ± 0.91	30.82 ± 4.70	0.719	No
EB	4.65 ± 0.31	4.36 ± 0.35	0.197	No
WVP	0.59 ± 0.04	0.59 ± 0.06	0.903	No

**Table 5:** Detailed FTIR peaks observed in Wheat cellulose (WC) and optimized wheat cellulose (OWC) film.

Band Region Wavenumber (cm^−1^)	Band Name	WC	OWC	Interpretation	References
3340–3400	O–H stretching; intra- and intermolecular hydrogen bonding	Broad O–H band	Broad band retained	Indicates retention of cellulose hydroxyl groups	[89,90]
2890–2925	C–H stretching of polysaccharide backbone	Present	Present	Characteristic cellulose band	[89,90]
1630–1650	H–O–H bending of absorbed water	Present	Present	Indicates hydrophilic nature of the cellulose matrix	[91]
1319, 1380, 1430	CH_2_ bending/deformation	Present	Present	Characteristic cellulose-associated vibration indicating cellulose-chain organization	[89,91]
1165	C–O–C asymmetric stretching	Present	Present	Characteristic polysaccharide fingerprint band retained	[92]
1000–1200	C–O stretching/C–O-related vibrations	Present	Present; slight spectral variation	Changes in position/intensity suggest reorganization of cellulose/formation of cellulose II during regeneration	[23,26,33]
~898	C1–H deformation/β-glycosidic linkage-associated vibration	Present	Present	Retention confirms preservation of the cellulose backbone after film formation	[93]
~1735	C=O stretching of acetyl/uronic ester groups associated with non-cellulosic components	Not prominent	Not prominent	Suggests effective removal of hemicellulose and other non-cellulosic components during alkali treatment and bleaching	[91]

**Table 6:** Summary of properties of optimized film (OWC) made with wheat cellulose.

Functional Property	Measured Value	Unit
Response variables: TS EB WVP	30.82 ± 4.7 4.36 ± 0.35 0.59 ± 0.06	MPa % 10^−10^ g.m^−1^ s^−1^ Pa^−1^
Physical properties: L* value, a*, and b*) a* value b* value WI YI ΔE Thickness	81.44 ± 0.70 −1.18 ± 0.31 4.79 ± 1.06 80.80 ± 1.19 8.40 ± 1.28 7.61 ± 0.80 0.06 ± 0.009	mm
Hydration property: Moisture content Water solubility Water absorption	13.63 ± 0.17 21.36 ± 4.26 42.89 ± 0.14	% % % at 120 min
Antioxidant activity: RSA IC_50_	10.3 0.33	% g/mL
Transparency	22.95 ± 0.65	mm^−1^ at 600 nm
95% Biodegradability	37	days

## Data Availability

The data presented in this study are available on request from the corresponding author. The data are not publicly available due to privacy restrictions.
